# Supplementary material for: Content-rich biological network constructed by mining PubMed abstracts
Source: BMC Bioinformatics. 2004 Oct 8;5:147. doi: 10.1186/1471-2105-5-147 (PMC528731; doi:10.1186/1471-2105-5-147)
Supplement: Additional File 2 — The original results of the above study (non-essential files are deleted to keep the file size under the limit set by BMC bioinformatics). [file 1471-2105-5-147-S2.bz2 › chilibotAdditionalFile2/dip10/500ID8041735E10665/html/TGFB2_TGFBR3.html]

 


 **TGFB2** and **TGFBR3** 
  
Found 27 abstracts in PubMed, retrieved 10.  
 

 What does Google say? 
 PDF only 
| .edu only 

---

**Interactive relationship** (e.g. stimulation, inhibition, etc)

**Stimulatory relationship**- Forced expression of betaglycan  [ **TGFBR3** ]  in CC myoblasts increases their responsiveness to TGF beta2  [ **TGFB2** ] , suggesting that it performs a TGF beta presentation function in this cell lineage.  Ref: 12399463 J Biol Chem, 2003
**Neutral relationship**- Ligand competition assays indicate that the relative affinities of recombinant soluble betaglycan  [ **TGFBR3** ]  for the TGF beta isoforms are TGF beta2  [ **TGFB2** ] >TGF beta3>TGF beta1.  Ref: 11256966 Biochem J, 2001
- The anti TGF beta potency of recombinant soluble betaglycan  [ **TGFBR3** ]  in vitro is 10 fold higher for TGF beta2  [ **TGFB2** ]  than for TGF beta1.  Ref: 11256966 Biochem J, 2001
- Compared with a commercial pan specific anti TGF beta neutralizing antibody, recombinant soluble betaglycan  [ **TGFBR3** ]  is more potent against TGF beta2  [ **TGFB2** ]  and similar against TGF beta1.  Ref: 11256966 Biochem J, 2001

**Non-interactive relationship** (e.g. studied together, co-existance, homology, etc.)

- Following abrogation of betaglycan  [ **TGFBR3** ]  expression with antisense oligodeoxynucleotide, embryonic lungs were relatively insensitive to TGF beta TGF beta2  [ **TGFB2** ]  0.5 ng ml and TGF beta1 20 ng ml, respectively, down regulated lung morphogenesis by 38 and 34% in control cultures.  Ref: 9739109 Mech Dev, 1998
- We therefore studied the expression of UPA, UPAR, IGF2R, ALK5, TGFBR2,  **TGFBR3** , ENG, ALK1, TGFB1,  **TGFB2** , and TGFB3 in a series of 14 pancreatic carcinoma cell lines.  Ref: 11408925 Int J Oncol, 2001
- Motoneurons were shown to synthesize the three proteins involved in transforming growth factor beta 2  [ **TGFB2** ]  signalling types I and II transforming growth factor beta receptor and betaglycan  [ **TGFBR3** ]  and to transport them anterogradely, where they were inserted into the axonal membrane and nerve terminal.  Ref: 10842018 Neuroscience, 2000
